# Relationship between obesity and health-related quality of life in children aged 7–8 years

**DOI:** 10.1186/s12955-018-0974-z

**Published:** 2018-07-28

**Authors:** Szabolcs Halasi, Josip Lepeš, Višnja Đorđić, Dejan Stevanović, Ferenc Ihász, Damjan Jakšić, Andrea Živković-Vuković, Milan Cvetković, Zoran Milić, Anita Stajer, Nevenka Zrnzević, Dragan Marinković

**Affiliations:** 1 Hungarian Language Teacher Training Faculty, University of Novi Sad, Strossmayer str. 11, Subotica, 24000 Serbia; 2 Faculty of Sport and Physical Education, University of Novi Sad, Novi Sad, Serbia; 3 Clinic for Neurology and Psychiatry for Children and Youth, Belgrade, Serbia; 4 Faculty of Education and Psychology, Eötvös Lóránd University, Budapest, Hungary; 5 Faculty of Education, University of Novi Sad, Sombor, Serbia; 6 Vocational School for Training Preschool Teachers and Sports Trainers, Subotica, Serbia; 7 Teacher Training Faculty, University of Priština-Kosovska Mitrovica, Leposavić, Serbia

**Keywords:** Obesity, HRQOL, Children, Overweight

## Abstract

**Background:**

The dramatic increase in the prevalence of obesity in developed and developing countries has become a major health care concern. Accordingly, there is growing recognition of the relationship between health-related quality of life (HRQOL) and obesity in the pediatric population. This study aimed to explore the relationship between HRQOL and different indicators of obesity in children aged 7–8 years.

**Method:**

In total, 182 children participated in this study (mean age 7.71 (0.29) years, 48.91% girls). To assess obesity, an InBody 230 analyzer was used to calculate body mass index (BMI) and body fat percentage (BFP). The proxy version of the KIDSCREEN-27 questionnaire was used to assess HRQOL.

**Results:**

Among boys, 17.2% were overweight and 4.3% were obese according to BMI, while in terms of body fat percentage (BFP), the corresponding percentages were 12.9 and 9.7%, respectively. Among girls, the prevalence of overweight and obesity was 11.2 and 9.0% by BMI and 10.1 and 7.9% in terms of BFP, respectively. The analysis of BFP showed a significantly higher score in normal weight boys than in obese boys in the Social Support & Peers domains (KW H-test = 10.472, *p* = 0.03), while in girls, there were no significant differences between weight categories and any HRQOL dimensions.

**Conclusion:**

Obesity at 7–8 years of age could negatively affect some HRQOL domains; in particular, obese boys may have low social support and peer functioning.

## Background

Excess weight and obesity are terms used to describe the accumulation of excess body fat, which can impair the individual’s health and well-being [1]. The main cause of obesity is an imbalance between energy intake and consumption; when caloric intake exceeds caloric consumption, the remaining energy is stored in the body as fat. According to the World Health Organization (WHO), obesity occurs in one of ten people worldwide [1, 2]. The prevalence of obesity is particularly high in developed and developing countries, even among children [2–4]. The prevalence of childhood obesity and overweight has increased sharply in the past decade and has presented a major global public health problem [5]. The results of the WHO-Collaborative Health Behaviour in School-aged Children (HBSC) survey showed that the prevalence of overweight and obesity was higher than 10% among school-age children in most countries, ranging from 7.6% in Latvia to 28.8% in the United States of America (USA) [5].

Obesity and weight gain are both associated with numerous health concerns including cardiovascular disease, type 2 diabetes, stroke and asthma [6]. The complications of obesity in childhood are significant and can include diabetes type 2 and hepatic steatosis. For example, approximately 40% of obese children have shown evidence of fatty liver changes using ultrasound imaging [7]. These effects are not only limited to individuals’ physical health, but also to their psychological and social health [8]. Health-related quality of life (HRQOL) is a subjective measure of an individual’s health and can be assessed using either generic or disease-specific instruments [9]. It was shown that obesity has a strong negative influence on HRQOL [9–15]. Obesity reduces life expectations [16] and leads to poor mental health as well [17]. Pediatricians believe that obesity in childhood or adolescence is a strong predictor of obesity in adulthood [1], which consequently affects HRQOL in adulthood [18]. Aside obesity, it has been also found that children with an increased amount of body fat have more negative perceptions of HRQOL than those with normal amounts of body fat [9, 12, 19].

There are no available data on the relationship between obesity and HRQOL in Serbian school children. This study was conducted to investigate obesity among primary school-aged children and to determine the relationship between obesity indicators and HRQOL. Greater body mass index (BMI) and body fat percentage (BFP) values were hypothesized to correlate with lower HRQOL in Serbian school children aged 7–8 years. From a clinical perspective, the relationship between HRQOL and BFP in children is unknown.

## Method

The population studied in this study included second-grade students aged 7–8 years, from Subotica (Vojvodina, Northern Serbia) and their parents. Stratified (geographically) random sampling was used in this cross-sectional study. Five schools were identified from the Hungarian Language Teacher Training Faculty and were randomly selected from Subotica on a proportional basis. Informed consent to participate in the study was obtained from children’s parents.

In total, 246 students were invited to participate, of which 182 students were assessed; 89 girls and 93 boys. The average age of the students was 7.71 ± 0.29 years.

All included students were healthy, with no somatic, neurological or psychiatric disorders at the time of the measurement.

Body weight and height, as well as the most representative measures of physical growth and development, were measured with standard measuring methods:

1. Body height (cm) was measured with a Martin anthropometer and
2. Body weight (0.1 kg) was measured with an InBody 230 body composition analyzer (Biospace Co., Ltd., Seoul, South Korea).

For research purposes, we selected two representative indicators of physical structure: BMI (kg/m^2^) and BFP (in percentages) both measured with an InBody 230 body composition analyzer (Biospace Co., Ltd., Seoul, Korea) [20]. BMI is the most common indicator used to assess obesity because it is an inexpensive and noninvasive measure of body heaviness. There is low observer error and good reliability and validity [21, 22], and BMI is thus widely recommended by experts as a simple and convenient measure of overweight for use during childhood [23]. BMI is calculated as the weight in kilograms divided by the square of height in meters. Bioelectrical impedance analyzers may be an accurate device for measuring BFP in children [24]. The literature on the validity and reliability of the InBody 230 indicate that the portable InBody 230 may be acceptable for calculating BFP as a measure of obesity [25]. The InBody 230 analyzer may thus be a valid method and has shown excellent reliability [26]. Obesity was defined according to the reference ranges for BMI [27] and for BFP [28].

The parent version of the KIDSCREEN-27 questionnaire was used to assess HRQOL, the Serbian version [29, 30]. The KIDSCREEN questionnaires are based on a multidimensional construct of quality of life, covering physical, emotional, mental, social, and behavioral components of well-being and functioning [29]. The questionnaires were adapted into Serbian in 2010, and the demonstrated intraclass correlation coefficients (ICCs) ranged from 0.38–0.63, indicating moderate to excellent agreement between children and parents when reporting HRQOL [30]. This agreement implies that the parent version could be used as a reliable and valid proxy measure of children’s HRQOL. The questionnaire contains five domains: 1) Physical Well-being (5 items), 2) Psychological Well-being (7 items), 3) Autonomy & Parent Relations (7 items), 4) Social Support & Peers (4 items), and 5) School Environment (4 items). The KIDSCREEN-27 requires 10–15 min to complete. To complete the questionnaire, parents respond to statements on a five-point Likert-type scale, indicating their level of agreement with the statement. The results are expressed in T-values ​​and percentages, which are obtained by using specific syntax formulated by the KIDSCREEN group in SPSS software [29]. Higher scores indicate higher levels of HRQOL in the domains assessed.

Descriptive statistics were calculated for all variables. For the relationship analysis between adiposity indicators Spearmann’s rank correlation was used. A Mann-Whitney U test was conducted to compare HRQOL between boys and girls, while Kruskal-Wallis H test was applied to examine the main effect of weight category on the HRQOL dimensions. For differences among groups, Mann-Whitney U test was applied to determine the differences between all pairs. Statistical significance was established at a level of *p* < 0.05. All analyses were performed using SPSS, PC program, version 20.0 (SPSS Inc., Chicago, IL, USA).

The study was conducted in accordance with the International Conference on Harmonisation of Good Clinical Practice, the Declaration of Helsinki and local ethical and legal requirements.

## Results

The mean values for anthropometric measures, body composition and HRQOL are shown in Table 1.

**Table 1:** Characteristics by gender

Variable	Male	Female
	Mean ± SD	Mean ± SD
Body Height (cm)	128.79 ± 6.11	127.28 ± 5.55
Body Weight (0.1 kg)	28.11 ± 5.31	27.43 ± 5.51
Body Mass Index (kg/m^2^)	16.84 ± 2.18	16.82 ± 2.50
Body Fat Percentage (%)	9.64 ± 8.02	22.52 ± 7.95*
Physical Well-being	55.77 ± 8.67	55.15 ± 9.24
Psychological Well-being	54.16 ± 11.13	56.48 ± 8.62*
Autonomy & Parent Relations	51.93 ± 9.46	52.83 ± 8.44
Social Support & Peers	54.50 ± 9.47	55.84 ± 8.00
School Environment	56.90 ± 9.40	58.70 ± 9.72

*SD*, standard deviation; *p < 0.05 boys vs. girls

In general, boys showed significantly lower BFP (19.64% vs. 22.52%) and lower scores on the HRQOL dimension of Psychological Well-being (54.16 vs. 56.48) than girls (*p* < 0.05). The prevalence of underweight and normal weight was 78.5% for boys and 79.8% for girls, based on the BMI, and 69.9 and 66.3% of boys and girls, respectively, as defined by BFP (Table 2).

**Table 2:** Weight categories

	Male (*n* = 93)		Female (*n* = 89)	
	n (%)	M ± SD	n (%)	M ± SD
Age (years)		7.8 ± 0.3		7.6 ± 0.3
Age group				
6.5 years	0 (0%)	0	1 (1.1%)	6.70
7 years	3 (3.2%)	7.21 ± 0.02	9 (10.1%)	7.19 ± 0.05
7.5 years	37 (39.8%)	7.54 ± 0.15	43 (48.3%)	7.48 ± 0.14
8 years	53 (57%)	7.95 ± 0.13	36 (40.4%)	7.99 ± 0.15
BMI-weight category (kg/m^2^)				
Underweight and normal weight	73 (78.5%)	15.90 ± 1.16	71 (79.8%)	15.82 ± 1.34
Overweight	16 (17.2%)	19.67 ± 0.80	10 (11.2%)	19.15 ± 0.74
Obesity	4 (4.3%)	22.65 ± 1.17	8 (9%)	22.72 ± 1.56
BFP-weight category (%)				
NR	5 (5.4%)	3.00 ± 0.00	5 (5.6%)	8.40 ± 2.39
Low	2 (2.2%)	8.95 ± 0.64	9 (10.1%)	13.93 ± 0.80
Mid	65 (69.9%)	17.63 ± 3.86	59 (66.3%)	21.61 ± 4.35
Upper	12 (12.9%)	27.52 ± 2.13	9 (10.1%)	32.62 ± 1.77
Obese	9 (9.7%)	35.24 ± 3.54	7 (7.9%)	38.36 ± 2.22

*NR* not rated, *BMI* body mass index, *BFP* body fat percentage

The relationship between BMI and BFP was calculated using Spearman’s rank correlations, suggesting a significant positive correlation (*r* = 0.76, *p* < 0.01).

**Fig. 1:**
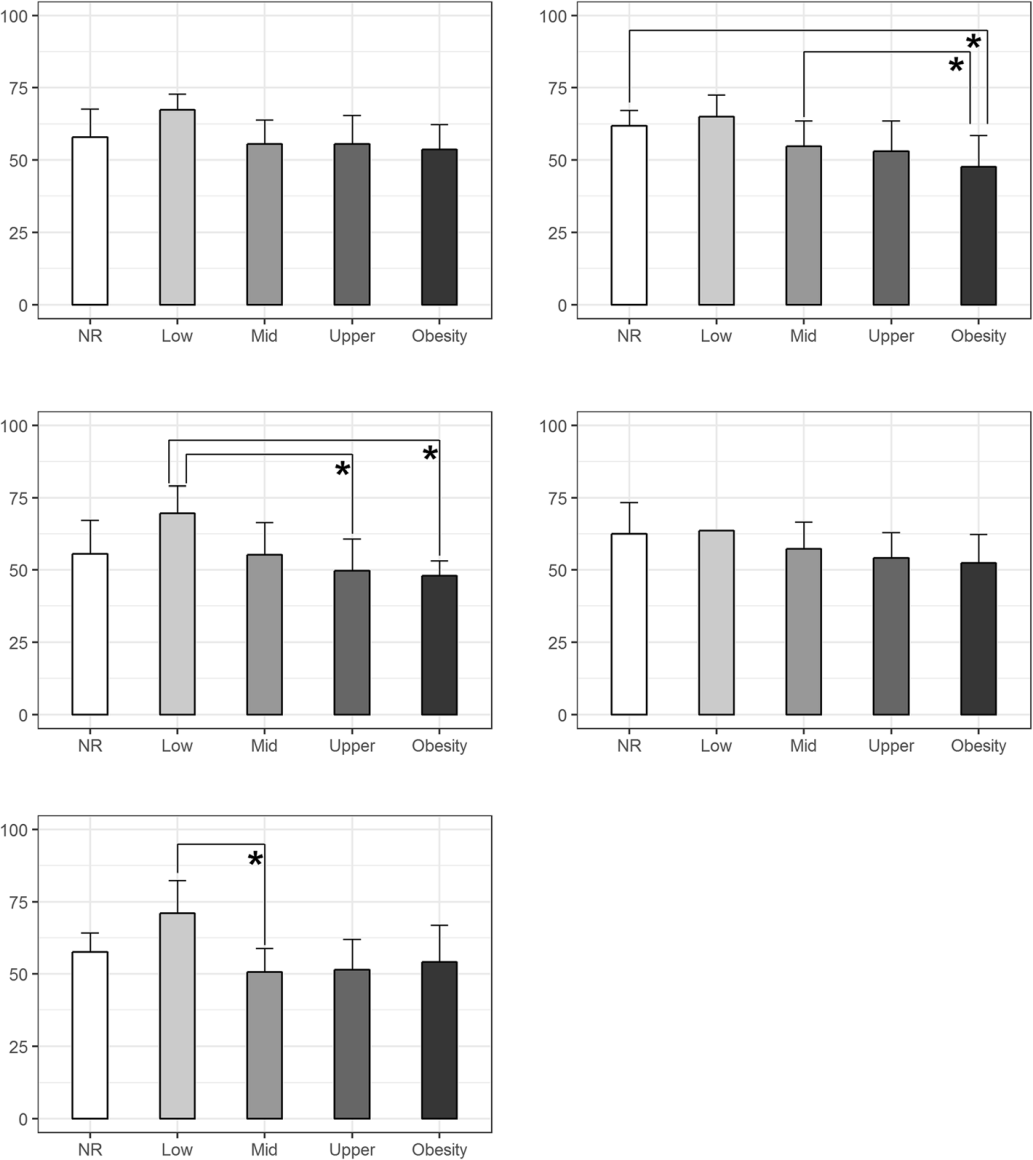
Differences between BFP weight categories in boys *p < 0.05

**Fig. 2:**
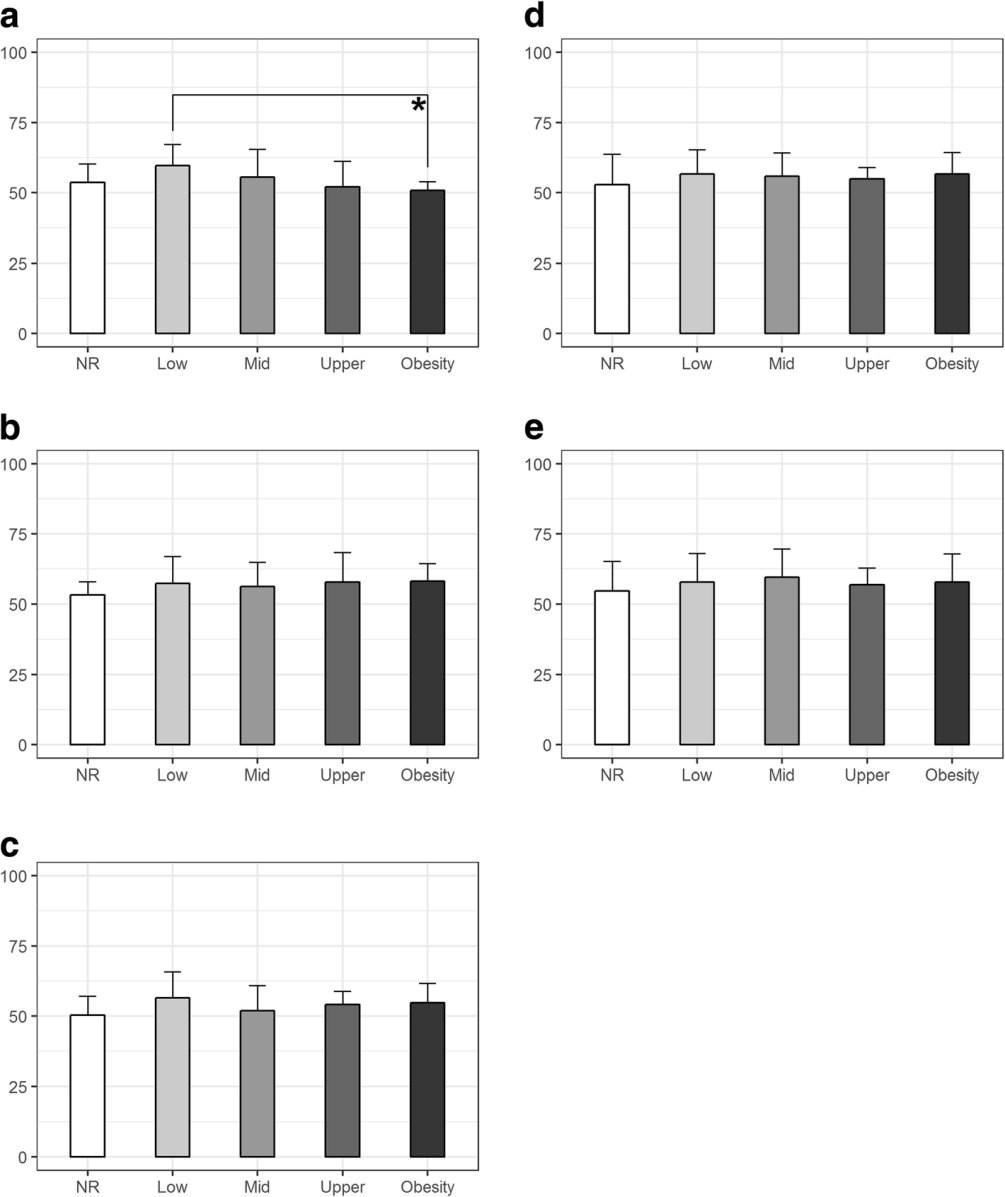
Differences between BFP weight categories in girls *p < 0.05

The differences in HRQOL and BMI between groups are presented in Tables 3 and 4, while the differences in HRQOL and PBF in Figs 1 and 2. The association between BFP and Social Support & Peers (*p* = 0.033) domains differed significantly in boys (Fig. 1).

**Table 3:** Differences between BMI weight categories in boys

	Underweight and normal *N* = 73	Overweight *N* = 16	Obesity *N* = 4		
Variable	M ± SD (MRank)	M ± SD (MRank)	M ± SD (MRank)	H	Sig.
Physical Well-being	56.12 ± 8.49 (48.38)	54.86 ± 10.09 (43.25)	53.07 ± 7.07 (36.75)	1.096	.578
Psychological Well-being	55.14 ± 11.74 (48.93)	50.26 ± 8.35 (38.47)	51.90 ± 5.39 (45.88)	2.002	.368
Autonomy & Parent Relation	51.84 ± 9.41 (46.37)	50.95 ± 9.74 (45.81)	57.39 ± 9.83 (63.25)	1.532	.465
Social Support & Peers	54.98 ± 9.51 (48.45)	54.33 ± 8.67 (46.59)	46.33 ± 10.35 (22.13)	3.685	.158
School	57.63 ± 9.45 (48.95)	54.37 ± 9.61 (40.31)	53.63 ± 6.44 (38.25)	1.826	.401

*M* mean *SD* standard deviation, *H* Kruskal-Wallis H test; *p < 0.05

**Table 4:** Differences between BMI weight categories in girls

	Underweight and normal *N* = 70	Overweight *N* = 10	Obesity *N* = 8		
Variable	M ± SD (MRank)	M ± SD (MRank)	M ± SD (MRank)	H	Sig.
Physical Well-being	55.46 ± 9.51 (46.38)	55.48 ± 8.53 (46.55)	50.07 ± 4.60 (30.81)	2.690	.261
Psychological Well-being	56.13 ± 8.76 (44.11)	58.15 ± 9.61 (50.65)	56.64 ± 6.98 (45.81)	.581	.748
Autonomy & Parent Relation	52.78 ± 8.90 (44.61)	51.71 ± 7.30 (41.85)	54.68 ± 6.34 (52.38)	.829	.661
Social Support & Peers	55.55 ± 8.34 (43.48)	57.62 ± 6.32 (51.05)	56.19 ± 7.25 (45.25)	.793	.673
School	58.29 ± 10.10 (44.48)	60.50 ± 6.85 (49.15)	58.49 ± 9.71 (44.44)	.301	.860

## Discussion

This is the only study to date that has evaluated HRQOL and obesity in primary school-aged children in Serbia. It was found that boys were slightly heavier on average than girls (28.03 kg vs. 27.43 kg), with the average body height of 128.77 cm for boys and 127.27 cm for girls, which agrees with the norms for the northern Serbia [31, 32]. The average BMI values of 16.85 kg/m^2^ in boys and 16.88 kg/m^2^ in girls indicated normal nutritional status, according to the WHO standard norms [33]. According to the reference values for BFP [28], 9.7% of boys and 8% of girls were in the obese category, and when considered BMI [27], 6% of boys and 9.1% of girls were obese. Girls had a higher percentage of body fat (22.26%) than boys (19.56%). These results are congruent with previous studies that suggested that gender differences in body fat content exist well before puberty [34–37]. Significantly higher body fat values are expected in girls because of their lower levels of physical activity, i.e., girls are engaged in games that do not require as much dynamic movement [32]. In contrast, boys show greater activity, leading to increased energy consumption, reduced subcutaneous fat tissue and increased muscle tissue [38, 39]. The differences in the body structure are evident from birth [40, 41], also the structure of the body changes during growth and development [42–45], when fat-free body mass continuously develops and increases in girls up to 15–16 years of age and up to 20 years of age in boys [41].

Considering the results related to HRQOL in the present study, the following was observed. First, there were significantly higher levels of psychological well-being among girls than among boys. This finding conflicts with the findings of a large European study using the same instrument, which showed that 8-year-old boys and girls had similar levels of quality of life, including psychological well-being [46]. However, the differences in quality of life become apparent during adolescence, when girls generally have lower levels of HRQOL. Second, girls had significantly higher BFP than boys in the study. A study evaluating obese children showed in primary school that third- and fourth-grade boys faced more physical and emotional difficulties than fifth- and sixth-grade boys, while the inverse was true for girls [47, 48].

Third, as measures of weight and indicators of obesity, it was observed that BFP rather than BMI as might be linked to HRQOL. Parents of children in different weight categories, including obesity based on BMI, reported similar levels of HRQOL across different domains. The difference in boys (underweight and normal vs. obesity) regarding to variable “Social Support & Peers” is large difference (8.65 points = 0.87 SD) [29], which is proved at PBF also. When different weight categories were compared using BFP, parents of obese boys reported significantly lower social support and peer functioning but higher autonomy and parent support than others. Obese boys had significantly lower scores on the social support and peer HRQOL subscales than normal weight children. This finding might be explained by the psychological consequences of obesity, which is one of the most stigmatizing conditions in childhood [49, 50]. In comparison to normal weight children, obese children are teased at least three times more often [51], and they are often perceived as lazy or stupid [52]. The self-concept of obese children might also be affected, and there is increased risk of social marginalization, which may further prevent obese children from developing social competences and gaining peer support [53].

A previous study with Latin American children showed that a higher BFP was associated with lower HRQOL scores, while BMI was not associated with any HRQOL dimension [47]. Direct measurements of body fat as a percentage of total weight provide a better index of adiposity and health risks than BMI [54, 55], which is insufficiently precise due to variations in fat-free mass in relation to height. Our findings also indicate that BFP may be more strongly associated with psychosocial functioning than BMI.

This findings indicate that high levels of BFP are followed by low levels of physical activity and a lower health status. Previous studies [55–57] obtained similar results, i.e., children with more weight had lower scores on HRQOL, and low scores were obtained for the dimensions Physical Well-being and Psychological Well-being. Energy consumption increases through physical activity, and a negative energy balance is created followed by reduction in BFP. Exercise improves general fitness, which can prevent a variety of diseases related to obesity [58]. Today’s trends, such as watching television and using other electronic media, reduce the time that children spend in movement while playing and increase the time spent on sedentary activities [59, 60]. The prevalence of overweight and obesity among children is increasing in many countries [50, 51], including Serbia [53]. One of the two most important reasons for this increase is thought to be the insufficient physical activity of children [54, 61, 62] combined with the consumption of high-calorie diets [55, 63]. Physical inactivity as early as preschool reflects a certain deviation from the norm, indicating physical or mental developmental problems or poor social adaptation [39]. Therefore, health policy makers should consider the potential additional benefits when promoting physical activity and healthy eating in Serbian schools. Additional studies on obesity treatment are needed to examine overweight or obese children and adolescents and to report the academic and cognitive as well as physical outcomes [64, 65].

There are several limitations of this study that should be considered. First, although the sample included 182 children age 7–8 years, the subgroups according to weight categories differed markedly in numbers, what affected the study power. It was found to be low to moderate between HRQOL dimensions and weight status indicators. Second, all children were sampled from one region, and the results might not be generalizable to other parts of Serbia. Third, the parent KIDSCREEN was the only instrument used, and despite its sound psychometric properties, self-reports were not included. This is an important aspect, because parents may over- or underestimate their child’s HRQOL [48]. Fourth, the study did not include older children, children with different health problems/disabilities, or children from a different sociodemographic and economic status. Finally, the cross-sectional design did not enable estimations of the true causal relationships between obesity and HRQOL.

## Conclusion

Obesity at age 7–8 years could negatively influence some HRQOL aspects; specifically, obese boys might have low social support and peer functioning. As an obesity measure, BFP may be a better indicator than BMI when studying HRQOL. The study needs to be replicated addressing the above limitations particularly to include other age groups and different variables in order to draw valid conclusions about of the HRQOL – obesity association.

## Abbreviations

BFP: Body fat percentage
BMI: Body mass index
HRQOL: Health-related quality of life
